# Yeast of Eden: microbial resistance to glyphosate from a yeast perspective

**DOI:** 10.1007/s00294-023-01272-4

**Published:** 2023-06-03

**Authors:** Dionysios Patriarcheas, Taizina Momtareen, Jennifer E. G. Gallagher

**Affiliations:** Department of Biology, West Virginia University, 53 Campus Drive, Morgantown, WV 26506 USA

**Keywords:** Glyphosate, EPSPS, Aro1, Dip5, Herbicide, Microbial resistance, Target-site resistance, Non-target-site resistance, In-lab evolutions, Genetic variation, Amino acid mimic

## Abstract

First marketed as RoundUp, glyphosate is history’s most popular herbicide because of its low acute toxicity to metazoans and broad-spectrum effectiveness across plant species. The development of glyphosate-resistant crops has led to increased glyphosate use and consequences from the use of glyphosate-based herbicides (GBH). Glyphosate has entered the food supply, spurred glyphosate-resistant weeds, and exposed non-target organisms to glyphosate. Glyphosate targets EPSPS/AroA/Aro1 (orthologs across plants, bacteria, and fungi), the rate-limiting step in the production of aromatic amino acids from the shikimate pathway. Metazoans lacking this pathway are spared from acute toxicity and acquire their aromatic amino acids from their diet. However, glyphosate resistance is increasing in non-target organisms. Mutations and natural genetic variation discovered in *Saccharomyces cerevisiae* illustrate similar types of glyphosate resistance mechanisms in fungi, plants, and bacteria, in addition to known resistance mechanisms such as mutations in Aro1 that block glyphosate binding (target-site resistance (TSR)) and mutations in efflux drug transporters non-target-site resistance (NTSR). Recently, genetic variation and mutations in an amino transporter affecting glyphosate resistance have uncovered potential off-target effects of glyphosate in fungi and bacteria. While glyphosate is a glycine analog, it is transported into cells using an aspartic/glutamic acid (D/E) transporter. The size, shape, and charge distribution of glyphosate closely resembles D/E, and, therefore, glyphosate is a D/E amino acid mimic. The mitochondria use D/E in several pathways and mRNA-encoding mitochondrial proteins are differentially expressed during glyphosate exposure. Mutants downstream of Aro1 are not only sensitive to glyphosate but also a broad range of other chemicals that cannot be rescued by exogenous supplementation of aromatic amino acids. Glyphosate also decreases the pH when unbuffered and many studies do not consider the differences in pH that affect toxicity and resistance mechanisms.

## Glyphosate inhibits the production of aromatic compounds

Excess exposure to any chemical is toxic to cellular metabolism either directly or excluding essential nutrients. The outcomes of chemical exposures can change depending on concentration and length of exposure. How cells respond to these chemicals provides insights into the regulation of cellular metabolism. The transport of chemicals into cells depends on the chemical properties and structure of these chemicals. Environmental exposures to synthetic chemicals are increasing over time and while active ingredients in herbicides are tested for toxicity, the effects of chronic exposure on humans and non-target organisms are a growing issue and are challenging to address with current approaches. Glyphosate is a broad-spectrum herbicide that inhibits EPSP synthase, an enzyme that converts shikimate 3-phosphate to 5-enolpyruvylshik**i**mate 3-phosphate (EPSP), a key step, in the shikimate pathway (also called the chorismate pathway) in plants, bacteria, and fungi. This leads to the depletion of chorismate, the precursor for all compounds containing aromatic rings, such as tryptophan, tyrosine, phenylalanine, pABA, and ubiquinone (Marbois et al. 2010) as well as metabolites from the TCA cycle (Zulet-Gonzalez et al. 2023). At lower doses, glyphosate itself does not kill plants but reduces their ability to grow and mount an effective immune response to infections, due to the aromatic precursors being building blocks for important molecules such as auxin, salicylic acid, and melatonin (Sauer et al. 2013; Pérez-Llorca et al. 2019), as well as structural compounds like lignin (Jalal et al. 2021). Both genetically modified crops and undesirable plants are sprayed with glyphosate-based herbicides (GBH). RoundUp^®^ Ready crops contain a bacterial ortholog of the glyphosate target (AroA), that does not bind glyphosate (Comai et al. 1983) and allows direct application to control undesirable plants. Metazoans, including humans, do not have the shikimate pathway**,** which reduces the acute toxicity of glyphosate exposure. Application to other crops such as wheat accelerates the drying process required before harvest. With glyphosate being the most popular herbicide, 181 million ha of glyphosate-resistant crops are grown (Duke 2018).

**Fig. 1:**
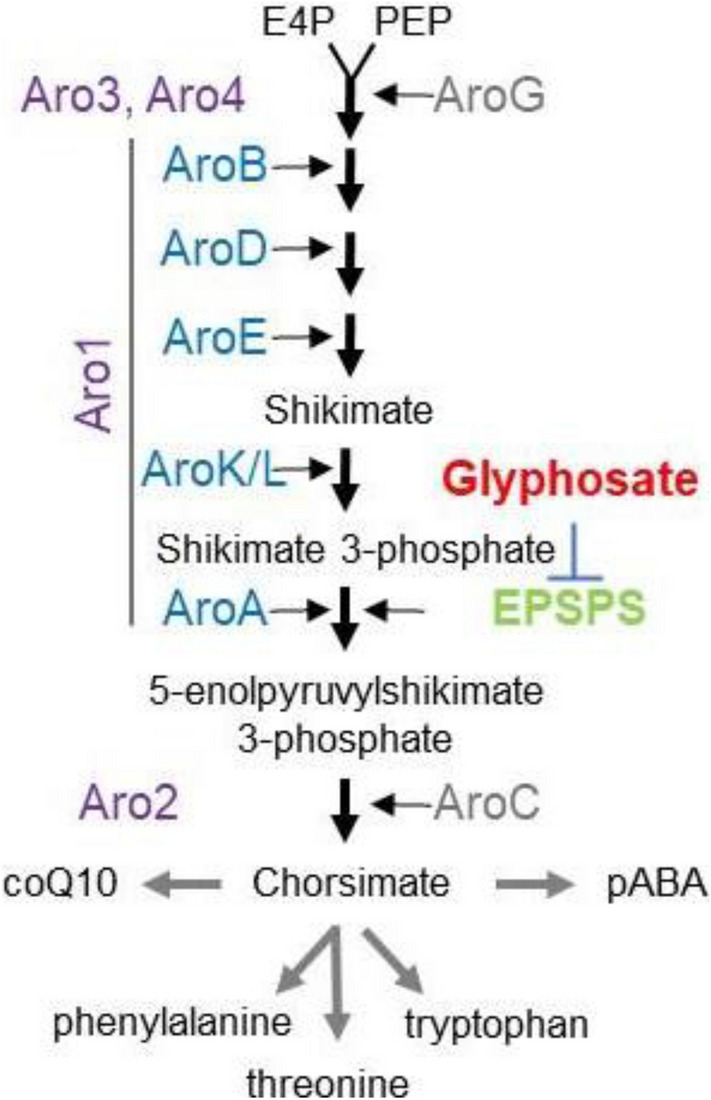
**Shikimate pathway** Phosphoenolpyruvate (PEP) and D-erythrose 4-phosphate (E4P) are the precursors to produce chorismate. Yeast (purple) and bacterial proteins (blue or grey) are labeled. Relevant compounds are in black. Glyphosate inhibits Aro1/AroA/EPSPS

The shikimate pathway is a multi-step process that converts phosphoenolpyruvate (PEP) and D-erythrose 4-phosphate (E4P) into chorismate (Fig. 1). Five of the seven enzymatic functions are present in the same yeast enzyme, Aro1, catalyzing steps 2 through 6. PEP can divert carbons away from the TCA cycle two different ways. It is a precursor of pyruvate and can be synthesized from malate, an intermediate of the TCA cycle. Using bacterial nomenclature, Aro1 encodes AroB, D, E, L, and A enzymatic functions converting 3-deoxy-D-arabino-heptulosonate-7-phosphate (DAHP) to 5-enolpyruvylshikimate-3-phosphate (EPSP) (Rong-Mullins et al. 2017; Wu et al. 2022). Aro2 then converts EPSP into chorismate. All phenolic compounds are derived from this pathway and are synthesized from coQ10, pABA, aromatic amino acids, or their precursors. Several other metabolites are made from shikimate pathway intermediates, including antivirals, dopamine, and salicylate by yeast and other organisms. Metabolic engineering of yeast can increase the production of these metabolites. L-Tyr and L-Phe allosterically inhibit Aro3 and Aro4 and prevent condensation of E4P and PEP (Suzuki et al. 1982).

## Genetic variation in aromatic amino acid biosynthesis generates large phenotypic diversity

In yeast synthetic media, nitrogen is provided as ammonium sulfate, but in the wild, any amino acid can serve as a nitrogen source. Natural genetic variation in Aro1 between a sake strain and a European wine strain links nitrogen consumption with growth (Cubillos et al. 2017). *ARO1* mutants are sensitive to many chemicals (Ayers et al. 2020). While further down the tryptophan branch, *trp1* mutants are sensitive to DNA damage (Brown et al. 2006), detergents (Schroeder and Ikui 2019), isobutanol (Liu et al. 2021), ethanol (Stanley et al. 2010), cold (Brachmann et al. 1998; Leng and Song 2016), pH (González et al. 2008), rapamycin (González et al. 2008), and MCHM (Ayers et al. 2020). These chemicals appear to generate oxidative stress, to which *trp* mutants are particularly sensitive. Within species, there is a wide range of genetic variation (Gallagher et al. 2014; Peter et al. 2018) that contributes to phenotypic diversity. However, some genetic diversity has a greater impact on phenotypic diversity than others. In particular, little genetic variation in transcription factors can have a large impact on phenotypes by changing the expression of hundreds of genes (Yvert et al. 2003; Gallagher et al. 2014). Low-diversity but high-impact genetic variation in these types of proteins are classified as master variators and are typically transcription factors (Gallagher et al. 2014). Genetic variation in three different yeast strains show several loci linked to glyphosate-based herbicides discussed below (Rong-Mullins et al. 2017; Ravishankar et al. 2020a).

## Glyphosate use and toxicity

Different countries have different acceptable glyphosate levels on foodstuffs, surface water, and blood sera levels. US and European guidelines for maximum exposures are 1.5 g mg/ kg/ day and 0.5 mg/kg/day, respectively. The lowest observed adverse effect level has been proposed to be 350 mg/kg/day, and the no observed adverse effect level is predicted to be 175 mg/kg/day in humans (Niemann et al. 2015). However, many studies expose animals to 0.5 to 50 mg/kg/day and detect physiological and molecular changes. While questions have been raised about the ability to extrapolate animal models to humans, human exposure to glyphosate is increasing over time, and the effects on health need to be studied (Mills et al. 2017; Gillezeau et al. 2019; Soukup et al. 2020; Huch et al. 2021; Grau et al. 2022). Mammals do not have the shikimate pathway but their microbiome consisting of bacteria and fungi do. Glyphosate-fed animals have significant changes to the gut microbiome population and metabolites (Mesnage et al. 2021). Mounting epidemiological studies have linked glyphosate exposure to cancers, neurodegenerative diseases, and sensory disorders (Myers et al. 2016). Therefore, the increasing levels of glyphosate exposure escalate the importance of determining how glyphosate resistance occurs, and what are the metabolic effects once it is transported into cells.

Glyphosate can be degraded by soil microbes by two different pathways. Amino-methyl phosphonic acid (AMPA) is easily detected in samples and often used as an indicator of glyphosate degradation. While glyphosate can degrade into AMPA and glyoxylate, an alternative pathway degrades it into sarcosine and phosphate depending on if the C-N or C-P bond is first cleaved. These pathways are complex and have different preferences in bacteria and fungi (reviewed in (Chen et al. 2022)). In soil microorganisms, the metabolism of glyphosate is affected by glyphosate’s ability to bind strongly to soil and metals (Sundaram and Sundaram 1997). The human microbiome presumably can also metabolize glyphosate. However, in humans, less than 1% of glyphosate is excreted as AMPA (Hori et al. 2003), and glyphosate is primarily excreted in the urine, but 30–40% of orally administered glyphosate crosses the intestinal wall (Brewster et al. 1991). Ingested glyphosate could be degrading through the C-P degradation pathway generating sarcosine which also is endogenously synthesized. It’s unknown if there is a natural source of AMPA and so it has become the metabolite most tracked to demonstrate glyphosate metabolism. While glyphosate is a water-soluble molecule, surfactants, primarily polyoxyethylene tallow amine (POEA), are added to commercial preparations to increase tissue permeability in commercial preparations. These “inert” ingredients have not been studied on their own, because they are proprietary and differ in the hundreds of different commercial preparations. Numerous formulations also contain heavy metals not listed on the labels (Defarge et al. 2018). Glyphosate is also a chelator and will bind divalent cations that influence its toxicity (Lanzarin et al. 2022). In animal models, orally administered GBH formulations increase glyphosate urine levels compared to the same concentration of pure glyphosate (Panzacchi et al. 2018). Similarly, yeast exposed to the same concentration of glyphosate are more sensitive to the commercial formulation than the pure chemical (Ravishankar et al. 2020b). The inactive ingredients in GBH increase the potency of glyphosate by increasing the penetration of glyphosate. By supplementing yeast with the downstream aromatic amino acids (WYF), the growth inhibition by blocking the production of chorismate is ameliorated (Rong-Mullins et al. 2017). However, there was genetic variation in the WYF rescue, hinting at other glyphosate targets in the mitochondria that are independent of the shikimate pathway (Ravishankar et al. 2020a). While some of the differences in gene expression were likely due to the surfactants, differences in internal concentrations of glyphosate could contribute to changes in gene expression or other unknown glyphosate targets (Ravishankar et al. 2020b).

## Yeast as a model to understand glyphosate toxicity

Extensive application of glyphosate has provided an example of evolution to anthropogenic effects on a wide range of organisms. *S. cerevisiae* is possibly the oldest domestic species and is closely associated with human fermentation activities, occupying a wide range of ecological niches. Yeast can be isolated from fruits, spontaneous fermentations of grain, and the human microbiome (Peter et al. 2018). Yeast are easily stored in freezer stocks and sampling historical collections provides insights into phenotypes. Like antibiotic resistance that existed in bacterial populations before the development of commercial antibiotics, the proportion of glyphosate resistance yeast is increasing over time (Barney et al. 2020). Yeast isolated from agricultural sources after the 1980s were the most resistant compared to forest or clinical sources. Glyphosate resistance from areas with known glyphosate exposure also correlated with length of exposure rather than recent high levels of exposure time (Barney et al. 2020). Areas from a state park with long-term glyphosate use to control invasive plants and maintain clearance for power lines had the highest glyphosate resistance (Barney et al. 2020). A former surface coal mine that was in the process of remediation was heavily sprayed with glyphosate the year before collection and had among the lowest measured glyphosate resistance in *S. cerevisiae* time (Barney et al. 2020). However, no *S. cerevisiae* were isolated along the Appalachian Trail, which is miles from human activity, highlighting its close association with human activities (Barney et al. 2020).

Glyphosate resistance can either be attributed to target-site resistance (TSR), namely mutations in the EPSPS gene leading to reduced glyphosate efficacy, or to non-target-site resistance (NTSR), which encapsulates all other genetic variations excluding EPSPS. Even though commercially available glyphosate-resistant crops possess a modified EPSPS and constitute an example of TSR, NTSR is reportedly the most widespread type of resistance to glyphosate (Powles and Yu 2010). In plants, glyphosate NTSRs remain largely understudied and mainly consist of mechanisms related to herbicide penetration, degradation, or mitigation of the collateral damage caused by the herbicide target inhibition (Délye 2013).

## Glyphosate formulations and toxicity

The effectiveness of glyphosate-based herbicides has made them the most heavily used weedkillers worldwide (Benbrook 2016). RoundUp, the most widely known and used GBH, has dominated the market since its inception in 1974. Two decades later, the introduction of genetically engineered RoundUp-resistant crops (better known as RoundUp ready) has led to a 15-fold increase in RoundUp use (Benbrook 2016). Since then, numerous other commercial formulations have been introduced, such as Compare-N-Save, Credit 41, WeedPro, Ranger Pro, and many more. In addition to glyphosate, GBH formulations contain solvents and adjuvants to enhance cell wall penetration (Brand and Mueller 2002). The additives are allegedly neutral, but studies have shown that when combined with glyphosate, such as in commercial formulations, they can induce undesirable effects in organisms (de Brito et al. 2019). The most common adjuvant in glyphosate-based herbicides is the surfactant POEA. According to the USDA, POEA makes up 15.4% of RoundUp. It has been shown that POEA-containing GBHs are about 100-fold more toxic to human cell lines than pure glyphosate and GBHs that lack POEA. Furthermore, there is a strong correlation between cytotoxicity and the concentration of POEA, but not glyphosate. Even formulations containing the same combination of glyphosate and POEA can have different levels of cytotoxicity, indicating the presence of other toxic formulants that vary across different GBHs (Mesnage et al. 2013).

One of the causes of GBH-induced cytotoxicity can be damage to the cell wall, because adjuvants such as POEA function by disrupting cell barriers. Sed1, the stress-induced cell wall protein-coding gene in yeast, is downregulated in GBH (one commercial formulation, Credit41)-sensitive cells but was found to have undergone gene duplication in GBH-resistant yeasts. This, along with the increased sensitivity of *sed1* mutants to GBHs, is indicative of a major role played by cell wall proteins in blocking the import of GBHs (Ravishankar et al. 2020b). Moreover, genotoxicity is also increased in cells exposed to GBH compared to pure glyphosate (Nagy et al. 2019). A study comparing DNA damage in human mononuclear white blood cells (HMWB) treated with pure glyphosate and three different GBHs (RoundUp Mega, Fozat 480, and Glyfos) found a significant increase in DNA breaks in the GBH group (Nagy et al. 2019). However, there is no conclusive evidence to affirm that the DNA damage is caused directly by the treatment and not a consequence of the cytotoxicity-induced cell death.

Numerous studies have concluded that GBHs harm non-target organisms over pure glyphosate. Spraying GBH on plants affects the organisms in the nearby ecosystem, such as animals near the field, fish in the water, and microbes in the soil (Wagner et al. 2013). POEA is widespread in and around agricultural farms that grow glyphosate-resistant crops (Tush and Meyer 2016). This is highly suggestive of POEA being incorporated into the food chain. GBH-resistant wild yeasts were found in several geographically diverse locations, including an organic farm that used to be a conventional farm before 1989 (Barney et al. 2020). This shows that the impact of GBH use on the surroundings lingers for decades after its degradation in the soil. A study on different laboratory yeast strains showed that a large number of mutations accumulated in cells that developed resistance upon treatment with the GBH, Credit41 (Ravishankar et al. 2020b). Transcriptomics from the same study comparing gene expression of Credit41-resistant strain (RM11) vs. sensitive strain (S288c) showed that the sensitive strain has differential gene expression in over 18 times as many genes as the resistant strain in minimal media, and over eight times as many genes in minimal media supplemented with the aromatic amino acids (Ravishankar et al. 2020b). When these cells were treated with Credit41 in the same growth conditions, the sensitive strain had gene expression variation in ten times more genes than the resistant strain (Ravishankar et al. 2020b). Among these differentially expressed genes, those that function in the synthesis of amino acids and secondary metabolites were downregulated, whereas genes involved in MAPK signaling, DNA replication, and the cell cycle were upregulated. Some of the upregulated cell cycle regulators, such as Nrm1 and Swi4, function specifically in the late G1 phase. This suggested that a block in G1 phase exit may be causing the cell cycle to arrest in Credit41-exposed cells. Indeed, 70% of the Credit-41-treated cell population underwent a G1 phase arrest, as demonstrated by flow cytometry. However, no such cell cycle arrest occurs in pure glyphosate-treated cells (Ravishankar et al. 2020b) but this may reflect the amount of glyphosate that enters the cells. Without detergents from GBH, pure glyphosate likely is imported less efficiently than glyphosate from GBH, reducing the intracellular levels of glyphosate with an equivalent level of glyphosate added. This further confirms that studies conducted with pure glyphosate do not mimic the effects caused by its commercially available forms.

## Mechanisms of glyphosate resistance

### Increased glyphosate efflux confers resistance

The flowering plant morning glory, *Ipomoea purpurea*, includes populations resistant to glyphosate intermixed with sensitive populations (Debban et al. 2015), making it a good candidate in uncovering mechanisms of resistance. The pattern of intermixed resistance and sensitivity lacking a geographical gradient suggests cases of independent evolution of resistance, as opposed to a derived trait from a common ancestor (Kuester et al. 2015). Genetic screens in the resistant populations found no nucleotide changes in the EPSPS sequence, indicating NTSR in *I. purpurea* (van Etten et al. 2020). Instead, they identified loci belonging to five genomic regions which included genes associated with glyphosate detoxification, including glycosyltransferases, responsible for conjugation, and ATP-binding cassette (ABC) transporters, responsible for transporting glyphosate into the vacuole. Evidence strongly supports genetic parallelism as a possibility for some of the more divergent genomic regions, as well as potential gene flow for the shared resistance-associated genomic region. Even though the genetic mechanisms of NTSR evolution are yet to be clarified, genomic regions associated with detoxification appear to bear particular significance, either through direct detoxification of the herbicide or through enabling oxidative stress management in *I. purpurea* (van Etten et al. 2020). Glyphosate-resistant plants produced fewer seeds and in the absence of glyphosate, had a fitness cost (Baucom and Mauricio 2004, 2008).

To uncover gene expression changes in herbicide-resistant *I. purpurea* transcriptomic comparisons between susceptible and resistant plants significantly downregulate 12 genes, 9 of which resembled kinases involved with cellular signaling (Leslie and Baucom 2014). The remaining genes were associated with cell growth arrest, cell wall biosynthesis, and saccharide catabolism. The upregulation of nine genes was also identified, among which was a transcript of the cytochrome P450 family, kinase transcripts linked to signaling, and microbial defense-associated factors. There was no difference in EPSP, differentiating *I. purpurea* from other glyphosate-resistant species (Leslie and Baucom 2014).

In a glyphosate-resistant *Echinochloa colona* (awnless barnyardgrass) population, RNA sequencing analysis linked resistance to two ABC transporter gene contigs (Pan et al. 2021). No amino acid substitutions were present in the resistant ABC genes, but the expression levels were significantly higher. Furthermore, overexpression of ABC-type C (ABCC) transporter orthologs in rice conferred resistance to glyphosate (Pan et al. 2021). This ABCC transporter is likely localized in the plasma membrane and functions as a glyphosate exporter, exporting glyphosate to the apoplast, the intercellular space, thus lowering intracellular glyphosate concentration. This proposed mechanism reduces intracellular toxicity; however, because it increases apoplast sequestration of glyphosate, it possibly results in higher glyphosate concentrations moved upwards through transpiration and accumulation in leaf tips and edges, which is consistent with observed leaf tip damage in rice seedlings exposed to glyphosate. Therefore, it is likely that in other plants as well, plasma-membrane-bound ABC-type transporters catalyze the export of glyphosate, contributing to genetic variation behind glyphosate resistance (Pan et al. 2021).

*S. cerevisiae,* a species with tremendous genetic diversity, occupies a wide range of niches, making it well suited for investigations of adaptation to new environmental stressors. Different yeast strains have different tolerances to glyphosate with agricultural isolates having the highest resistance (Barney et al. 2020). Exploiting the natural genetic variation found genes responsible for glyphosate tolerance. To address the genetic variation in glyphosate tolerance, Quantitative Trait Loci (QTL) analysis was carried out between two strains that demonstrated the greatest divergence in phenotypic response to glyphosate (Rong-Mullins et al. 2017). QTL was carried out in three different conditions between S288c, a laboratory strain, and YJM789, a clinical isolate. Yeast grown in minimal media (YM) would require activity from the shikimate pathway, because no aromatic amino acids are added. Yeast grown in YM + WYF would permit the identification of NTSR, because supplementation with WYF would bypass the inhibition of the shikimate pathway. In rich media (YPD), we predicted that NTSR would also be identified because WYF is present in the media and fourfold more GBH was needed to reduce growth comparable to YM (Rong-Mullins et al. 2017). Interestingly, there was no evidence of genetic variation within the *ARO1* gene in *S. cerevisiae*, the ortholog of EPSPS, suggesting non-target-site resistance. In YPD, one of the loci of interest was *PDR5*, encoding an ABC transporter, a family protein that has been well associated with highly variable drug resistance (Guan et al. 2010). The allele from S288c conferred resistance to GBH compared to YJM789, a human microbiome isolate. However, strains expressing *PDR5*^*S288c*^ were primarily resistant in rich media when aromatic amino acids are present but there was a slight effect on minimal media (Rong-Mullins et al. 2017) Another QTL between S288c and RM11, a GBH-resistant agricultural isolate, did not identify the *PDR5* locus contributing to genetic variation in glyphosate growth but identified other loci (Ravishankar et al. 2020a)*.* The highly polymorphic sequence of the Pdr5 transporter on the nucleotide and amino acid level enables a wide range of chemicals to be transported (Guan et al. 2010). Between strains YJM789 and S288c, there is a 5% amino acid sequence difference in Pdr5 (Guan et al. 2010), but no particular polymorphism was associated with glyphosate sensitivity (Rong-Mullins et al. 2017). Overexpression of the yhhS gene *E. coli* confers resistance to glyphosate through reduced accumulation inside the cell. The yhhS gene is a member of the MFS transporter family, which also includes ABC transporters, suggesting that overexpression leads to an increase in glyphosate export in both *E. coli* and *Pseudomonas* (Staub et al. 2012). Deletion of *PDR5* leads to increased sensitivity to glyphosate and in accordance with its known role in drug transport, it is suggested that it contributes to the export of glyphosate across different species.

## Glyphosate influx inhibition as a mechanism to glyphosate resistance

**Fig. 2:**
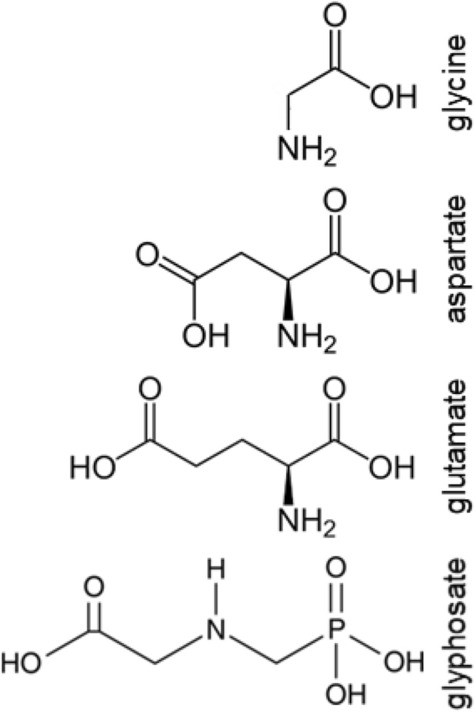
**Chemical structures of amino acids and glyphosate** Glyphosate is glycine analog that is likely transported through the same permease as aspartate and glutamate. Glyphosate has similar size, shape and charge distribution as aspartate and glutamate, suggesting that glyphosate is an amino acid mimic

In YM, the variation in glyphosate resistance mapped to an amino acid permease, Dip5 (Rong-Mullins et al. 2017). While *DIP5* deletion increased glyphosate resistance, expression of the resistant allele further improved the growth of yeast on glyphosate (Rong-Mullins et al. 2017). Dip5 localization to the cell membrane is decreased by the addition of aspartate or glutamate (Hatakeyama et al. 2010; O’Donnell et al. 2013), and excess glutamate or aspartate relieved growth inhibition of all yeast tested in response to glyphosate (Rong-Mullins et al. 2017). Even though glyphosate is an analog of glycine, the phosphonate group changes the size and charge distribution to resemble glutamate and aspartate (Fig. 2). Glyphosate acting as an amino acid mimic provides a mechanism of transport into the cell using D/E transporters. During the search for the mechanism of glyphosate, others research groups had proposed that glyphosate affected glutamate in plants (Killmer et al. 1981; Nafziger et al. 1983) but were overshadowed by the discovery of EPSPS as the TSR (Steinrücken and Amrhein 1980; Comai et al. 1983). Given that glyphosate is transported as a D/E mimic, it is likely that other D/E-requiring enzymes also are negatively affected by glyphosate.

The remarkable bacterial biodiversity underlies varying levels of sensitivity and adaptation to glyphosate. Firmicutes appear to be more resistant to glyphosate compared to proteobacteria, with actinobacteria demonstrating the most sensitivity. A pattern of bacterial habitats also indicates that free-living prokaryotes appear more resistant, as opposed to facultative host-associated or intracellular bacteria. Similar to yeast, the addition of D/E to *B. subtilis* rescues growth inhibition from glyphosate exposure (Wicke et al. 2019) and point mutants and deletions of GltT, a high-affinity D/E transporter, confer glyphosate resistance. GltT has no structural homology to Dip5 but is a bacterial D/E transporter. Across two different kingdoms of life, glyphosate is transported into cells using the D/E transporter, and deletion of the transporter or addition of excess D/E rescues glyphosate-induced growth arrest (Rong-Mullins et al. 2017; Wicke et al. 2019). The same phenomenon likely occurs in humans. While humans do not have the shikimate pathway, other metabolic pathways use D/E, particularly in the mitochondria, such as the synthesis of nearly all amino acids (directly and indirectly), heme, nucleotides, glutathione, and GABA biosynthesis, as well as the TCA and urea cycle. Inhibition of yeast growth is partially rescued when the aromatic amino acids are added but not to the same extent as adding D/E, suggesting other glyphosate targets (Rong-Mullins et al. 2017). Therefore, it seems that members of this protein family are involved in glyphosate efflux both in plants, bacteria, and yeast. Recent work has also shown that AimA encodes for a high specificity glutamate transporter in *B. subtilis* (Krüger et al. 2021). However, this was not identified in the screening by Wicke (Wicke et al. 2019) which poses the question of whether AimA has a higher specificity for glutamate with GltT mediating the transport of glyphosate, as well as glufosinate, another herbicide that inhibits glutamine synthetase (Hertel et al. 2021).

Glyphosate is likely imported by D/E transporters using amino acid mimicry. Therefore, other proteins that use D/E could be affected by glyphosate and alter metabolism. The malate-aspartic acid shuttle generates cytoplasmic glutamate while moving protons from NAD^+^ into the mitochondria effectively importing NADH [reviewed (Broeks et al. 2021)]. Malate is an intermediate in the citric acid cycle. While high levels of N-acetylglutamate in the mitochondria inhibit ammonia metabolism through the urea cycle. Additionally, cycling between alpha-ketoglutarate and glutamate moves an amino group through multiple metabolic pathways. A side effect of glyphosate inhibiting shikimate is increased levels of PEP, one of the necessary components to build shikimate. Citric acid cycle intermediates increase in glyphosate-resistant *Amaranthus palmeri* in a dose-dependent manner while increased levels were detected in sublethal exposed glyphosate-resistant varieties (Zulet-Gonzalez et al. 2023). The glyphosate-resistant *A. palmeri* have amplified the EPSP loci; however, the entire genome was not sequence so it is unknown what other compensatory mutations occurred (Fernández-Escalada et al. 2016). The glyphosate’s effect on mitochondria could be two-fold: glyphosate could directly block carbon flow through the shikimate which would then increase PEP levels, a pyruvate precursor which would increase intermediates of the citric acid cycle while glyphosate as a D/E mimic in the mitochondria could overwhelm aspartate mitochondria export or act as a competitor for aspartic acid import.

## Further genetic variation contributing to glyphosate resistance

**Fig. 3:**
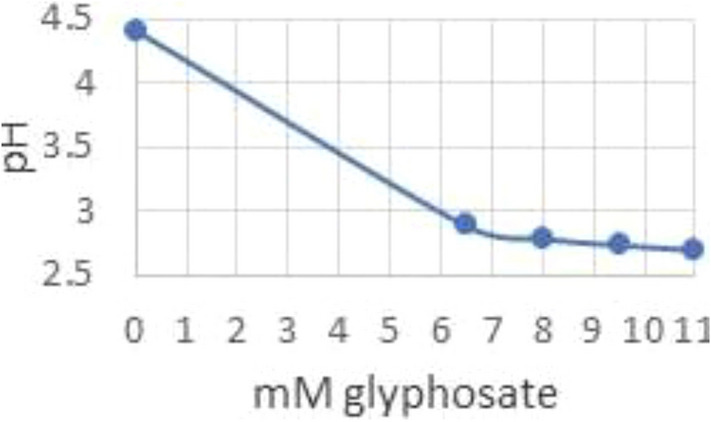
**pH of yeast minimal media with pure glyphosate added at different concentrations**

With genetic variation being a powerful tool in understanding glyphosate modes of action, In Lab Evolution (ILE) was employed to induce selection and resistance to glyphosate. Two sensitive and two resistant yeast strains were grown in the presence of a commercial glyphosate-based herbicide in minimal or rich media, with herbicide only or herbicide and aromatic amino acids. Samples were then passaged six times by transferring 1% to fresh media to induce adaptation. Resistant populations were isolated and sequenced. A total of 148 genes were identified to have accumulated at least 1 non-synonymous polymorphism in the coding region (Ravishankar et al. 2020b). Copy number variation was also assessed and indicated a variety of pathways affected through duplications, some specific to the condition of growth and the strain. Genomic loci that were amplified contained *ARO1*, *TMS1* which encodes a Pdr5-interacting vacuolar protein, and *VMA2*, which encodes a subunit of the vacuolar H + ATPase that maintains intracellular pH (Ravishankar et al. 2020b). Plants in the lab and wild often amplify EPSP in the genome which correlates with glyphosate resistance (Shah et al. 1986; Widholm et al. 2001; Gaines et al. 2010; Fernández-Escalada et al. 2016). Yeast are tolerant to low pH and actively pumps protons into the media to drive the secondary transport of nutrients across the cell membrane (Eskes et al. 2018). While acidification of the vacuolar is critical for stress response (Milgrom et al. 2007; Ayers et al. 2020) and amino acid homeostasis (Shimazu et al. 2012). Glyphosate’s pKa is acidic (Borggaard and Gimsing 2008) and glyphosate reduces the pH yeast minimal media from 4.5 to 2.8 pH when 8 mM glyphosate (Fig. 3). Whereas GBHs are buffered, and the pH does not decrease. Glyphosate also drops the pH below 3 in zebrafish media when at similar concentrations to the yeast system (Schweizer et al. 2019). The effects on zebrafish embryos were more pronounced when the system was not buffered (Schweizer et al. 2019). Metal chelation is also affected by pH (Glass 1984). From transcriptomic data, *CTR1* and *FET4* are downregulated when exposed to GBH. (Ravishankar et al. 2020b). Of the hundreds of commercial formulations, one GBH, Credit41, also contains magnesium, calcium, and potassium in addition to the surfactants (Ravishankar et al. 2020a). Glyphosate chelates divalent cations such as copper, zinc, manganese, magnesium, and calcium as the pH increase with copper binding at the lowest pH and 100% complexed with glyphosate at pH 4 Madsen et al. 1978). Glyphosate’s predicted Fe^+2^ binding is 6.87 which is between zinc at 8.74 and manganese at 5.47 but Fe^+3^ binding is 16.09 which is higher than copper at 11.93 (Madsen et al. 1978; Duke et al. 2012). Fe + ^3^ converts to Fe + ^2^ as pH increases with equal concentration at pH 2.5. The metal binding is correlated with pH and pKa of the first hydrolysis of the metal (Duke et al. 2012). Many studies in numerous models do not account for the drastic drop in pH induced by the addition of glyphosate to the media. Aquatic organisms are particularly sensitive to low pH.

## Perspective

Future studies should take into account how glyphosate changes pH, glyphosate complexed with different metals, the impact of buffering by GBH, and the likely effects of glyphosate on NSTR through amino acid mimicry because of structural similarities with D/E. Through In-Lab evolutions, natural genetic variation and transcriptomics have found functions associated with the copy number variants including mitochondrial maintenance, biosynthesis, DNA damage repair, spindle formation, metal transport, cell wall, and cell membrane (Ravishankar et al. 2020b). This is yet another example of mutations occurring outside the target EPSPS gene but in this case, resistance was not localized within a single gene or a single pathway, but rather a collection of loci that potentially contributes to NTSR. The problem of glyphosate resistance in fungi extends past environmental exposure but also into development of new antifungal targets. Fungal infections are notoriously difficult to treat. Fungi retained additional amino acid biosynthetic pathways including branch chain amino acids that are not in humans and are potential targets but potential resistance would need to be studied (Kuplińska 2021).

## Data Availability

All data discussed are available from cited sources within the text. Specifically RNAseq from yeast treated with GBH are available on Figshare: 10.25387/g3.12132924.
